# The response of mental health professionals to clients seeking help to change or redirect same-sex sexual orientation

**DOI:** 10.1186/1471-244X-9-11

**Published:** 2009-03-26

**Authors:** Annie Bartlett, Glenn Smith, Michael King

**Affiliations:** 1 Division of Mental Health, St George's University of London, London, UK; 2 Department of Mental Health Sciences, University College Medical School, University College London, London, UK

## Abstract

**Background:**

we know very little about mental health practitioners' views on treatments to change sexual orientation. Our aim was to survey a representative sample of professional members of the main United Kingdom psychotherapy and psychiatric organisations about their views and practices concerning such treatments.

**Methods:**

We sent postal questions to mental health professionals who were members of British Psychological Society, the British Association for Counselling and Psychotherapy, the United Kingdom Council for Psychotherapy and the Royal College of Psychiatrists. Participants were asked to give their views about treatments to change homosexual desires and describe up to five patients each, whom they has treated in this way.

**Results:**

Of 1848 practitioners contacted, 1406 questionnaires were returned and 1328 could be analysed. Although only 55 (4%) of therapists reported that they would attempt to change a client's sexual orientation if one consulted asking for such therapy, 222 (17%) reported having assisted at least one client/patient to reduce or change his or her homosexual or lesbian feelings. 413 patients were described by these 222 therapists: 213 (52%) were seen in private practice and 117 (28%) were not followed up beyond the period of treatment. Counselling was the commonest (66%) treatment offered and there was no sign of a decline in treatments in recent years. 159 (72%) of the 222 therapists who had provided such treatment considered that a service should be available for people who want to change their sexual orientation. Client/patient distress and client/patient autonomy were seen as reasons for intervention; therapists paid attention to religious, cultural and moral values causing internal conflict.

**Conclusion:**

A significant minority of mental health professionals are attempting to help lesbian, gay and bisexual clients to become heterosexual. Given lack of evidence for the efficacy of such treatments, this is likely to be unwise or even harmful.

## Background

A combination of social factors, legal sanctions against homosexual behaviour and the rise of behaviourism in the 1950s led to the development of psychological treatments to make homosexual men heterosexual. The application of these treatments rose to a peak in Britain in the 1960s and 70s [1]. In an earlier paper, we examined the motivations and experiences of 30 mental health professionals who had developed and practised these treatments from the 1950s to the 1980s [2]. These oral history data showed that, even at the time that they developed the treatments, many of the practitioners were ambivalent about them. Over the years they gradually became aware that the medicalisation of homosexuality and attempts to change it were influenced by cultural stereotypes and social prejudice. These data, however, only concerned specialists in this area. We know very little about how widely such developments were taken up by mental health professionals across the country and the extent of their use today. Thus, our aim in this study was to explore the views and practice in relation to sexual orientation of a wider field of mental health practitioners in the United Kingdom (UK).

## Methods

The study was approved by the former Royal Free Hampstead NHS Trust's Ethical Practices Subcommittee.

### Participants

We recruited participants for a questionnaire study from the then current (2001/2002) membership directories of the British Psychological Society (BPS) (approximately 2,047 members); the British Association for Counselling and Psychotherapy (BACP) (4,500 members); the United Kingdom Council for Psychotherapy (UKCP) (5,400 members); and the Royal College of Psychiatrists (RCP) (4,841 members). These organisations take a diversity of approaches to the treatment of mental health problems. In a study of this sort it is difficult to estimate a suitable sample size. We aimed to recruit up to 1600 participants in order to have sufficient power for our main comparisons between practitioners in the four organisations, and between those who had attempted to change sexual orientation and those who had not.

### Random selection

We selected 462 members randomly from each organisation, making 1848 in all. To avoid over-sampling in major conurbations such as London, we assigned each member of each list a number and then randomly selected by computer, weighting the selection to the proportion of the UK population in that region (Office of National Statistics: Government Office Regions 2003). Cross-referencing eliminated members affiliated to more than one organisation.

### Questionnaire

We posted the questionnaire in July and August 2002 with a covering letter explaining the nature and purpose of the study. Non-responders were followed up once and last questionnaires were received by April 2003. Participants returned the questionnaire and a stamped addressed postcard separately in order for us to keep responses anonymous and yet be able to remind non-responders. After collecting standard data on age, gender and profession, an introductory question addressed how the therapist would respond to a client who consulted them to change or redirect their sexual orientation. The second question asked whether they had ever assisted a client to reduce or change their same-sex desires. If this had never been the case, the respondent did not answer further. Those who continued answered a series of closed and open questions on their assessment and experience of up to five such clients, including the client's age and sex, the referral source, where treatment took place, what kind of treatment was offered and for how long clients were followed up. Those participants who gave information on a client of this sort were asked whether a service should be available to people who wanted to change from homosexual to heterosexual. We did not pose this question to the full sample as we believed their responses would be hypothetical given they had no personal experience of such treatments. No words to describe same sex attraction and behaviour are value free. Throughout the questionnaire we used the adjectives homosexual and lesbian not because we believe these were particularly apposite but in order to make sure that the therapists (for whom these terms might be most familiar and least political) would be clear about our meaning.

### Analysis

We used descriptive statistics to examine answers to each structured question and multivariable logistic regression to examine the characteristics of therapists who described treating clients to reduce or change their same-sex desires. The quantitative data were analysed using Stata release 9. The qualitative data arising from the open questions on therapists' work with clients seeking help about their sexual orientation were entered into a thematic content analysis. All qualitative data were considered in relation to the aims of the study in accordance with the applied nature of the wider study [3]. The volume of material generated in the questionnaire allowed for manual analysis. Initial indexing of data on individual patients treated by respondents was undertaken by a non-clinical researcher (GS) and then reviewed and refined by a senior clinician (AB) resulting in consensus about a smaller number of refined, descriptive categories. Data related to principles of treatment were read and reread and descriptive categories generated to elucidate similarities and differences between and within professional groups. Allocation of categories was limited by the small volume of text generated any individual respondent. Respondents' comments included in this paper are chosen as key illustrations of main emerging themes [3].

## Results

### Response rates and characteristic of respondents

Of 1,848 questionnaires posted, 1,406 were returned; 73 of these were returned from unknown addresses or were sent back by relatives where the participants had died, and in five returns we could not determine the respondent's affiliation. This left, 1,328 returns of which 351 (76%) came from members of the BPS, 357 (77%) the BACP, 331 (72%) the UKCP and 289 (63%) from the Royal College of Psychiatrists. There was a small degree of mismatch between participants' descriptions of their main professions and their affiliate societies (table 1). Participants' mean age was 51 years, two thirds were women, and mean years in practice was 15 (table 2).

**Table 1:** Main profession and affiliation

	Psychologist	Counsellor	Psychotherapist	Psychiatrist	Total
BPS	319	25	9	13	366

BACP	7	253	98	-	358

UKCP	30	6	268	-	304

RCP	2	-	2	277	281

All	358	284	377	290	1309

Missing = 78

BPS: British Psychological Society; BACP: British Association for Counselling and Psychotherapy; UKCP: United Kingdom Council for Psychotherapy; RCP Royal College of Psychiatrists

**Table 2:** Characteristics of professionals

	BPS N = 351	BACP N = 357	UKCP N = 331	RCP N = 289	Total N = 1387
Women^25 ^(%)	212 (62%)	296 (84)	251 (76)	93 (32)	852 (65)

Age^68 ^(sd)	50 (10)	54 (8)	53 (8)	44 (9)	51 (10)

Mean years in practice^4 ^(sd)	17 (10)	11 (6)	14 (7)	17 (10)	15 (9)

BPS: British Psychological Society; BACP: British Association for Counselling and Psychotherapy; UKCP: United Kingdom Council for Psychotherapy; RCP Royal College of Psychiatrists

Number in superscript = number missing; sd = standard deviation

### Attitudes to treatment

Participants gave one or more responses to an introductory question on how they would manage a hypothetical client seeking treatment to change his or her sexual orientation. Only 55 (4%) reported that they would attempt to change the client's sexual orientation (table 3). Members of the BACP were most likely to counsel them to accept their sexuality and least likely to assist them to change their sexuality. Psychiatrists were most likely to refer on to other colleagues who might help them to adjust to the sexuality. Five hundred and one respondents chose to write a comment in which the majority was interested in understanding the client's motivations to seek treatment and exploring those with them (table 3).

**Table 3:** Introductory question on how they would manage a client seeking to change or redirect their homosexuality

	BPS	BACP	UKCP	Psychiatrist	Total	Statistic
Assist them to accept their sexuality	182(52)	225(68)	188 (57)	136 (47)	731 (55)	Chi2 = 18.7 P = 0.000

Assist them or give them treatment to change their sexuality	19 (5)	12 (3)	15 (5)	9 (3)	55 (4)	Chi2 = 2.9 NS

Refer them to another colleague who has more experience of assisting men and women to accept themselves	93 (27)	49 (14)	41 (12)	127 (44)	310 (24)	Chi2 = 111.9 P = 0.000

Refer them to a colleague who may help them change or redirect their homosexual or lesbian feelings	24 (7)	39 (11)	23 (7)	45 (16)	131 (10)	Chi2 = 17.8 P = 0.000

Assist them to gain more effective control of their sexual feelings with a view to reducing personal and/or social difficulties	121 (34)	120 (34)	112 (34)	100 (36)	456 (34)	Chi2 = 0.34 NS

Other	131 (37)	136 (38)	155 (47)	69 (24)	491 (37)	Chi2 = 35.3 P = 0.000

(n) column per cent – adds to more than 100% as more than one answer could be selected.

BPS: British Psychological Society; BACP: British Association for Counselling and Psychotherapy; UKCP: United Kingdom Council for Psychotherapy; RCP Royal College of Psychiatrists

### Reducing or changing same-sex attraction

Two hundred and twenty two professionals (17%) reported having treated at least one client/patient in order to reduce or change his or her homosexual or lesbian feelings [72 (21%) BPS; 58 (16%) BACP; 59(18%) UKCP; and 33 (11%) RCP, Chi2 = 9.8, p = 0.02]. In a logistic regression (1249 observations), men were more likely than women (odds ratio OR 1.7, 95% confidence intervals CI 1.2, 2.4); older more likely than younger professionals (OR per year of age 1.02, CI 1.01, 1.04); and BPS members (OR 2.1, CI 1.3, 3.3) and UKCP members (OR 1.8, CI 1.1, 3.0) more likely than members of the Royal College of Psychiatrists) to have helped a client in this way.

### Details of treatment described

These 222 professionals gave details on at least one homosexual patient that they had helped to become heterosexual. A total of 413 clients were described (table 4) of whom 138 (35%) were referred by GPs, 88 (22%) by other professionals and 172 (43%) referred themselves (in 15, no referral source was given). Of the patients described, 33 (8%) were seen between 1963 and 1980 while the remainder (373) was referred after 1980 (seven professionals were unable to give the year of consultation). The mean number of clients described per calendar year ranged from1 to 5 (note that participants could only describe up to five clients), with 17 clients receiving treatment before 1973, and 24, 70 and 295 treated in the decades 1974–1983, 1984–1993 and 1994–2003 respectively (in 6 cases the year was not given). Although these figures may be biased towards recency by the relative age and memories of the therapists, it would seem there has been little or no decline in numbers treated over recent decades. The reasons given for the client seeking help could be summarised as confusion about sexual orientation (236, 57%) social pressures including the family (59, 14%), mental health difficulties (45, 11%), religious beliefs (28, 7%), gender confusion (15, 4%), legal pressures (14, 4%), heterosexual relationship difficulties (9, 2%), and as victims of abusive relationships (8, 2%). Two hundred and thirteen (52%) clients were seen in private practice, 162 (40%) in NHS practice and 33 (8%) in other (e.g. voluntary service) practice (in five instances the service was not specified). Two hundred and sixty five clients (66%) received counselling, 56 (14%) psychotherapy, 59 (15%) behaviour therapy, 10 (2%) medical treatment, 6 (2%) other treatment and 5 (1%) were referred on (professionals did not describe the treatment in 12 clients). In 117 cases (28%) the therapist did not follow-up the patient beyond the end of treatment. Median follow-up for the remainder was eight months (interquartile range 4–18).

**Table 4:** Details of patients treated

		BPS N = 351	BACP N = 357	UKCP N = 331	RCP N = 289	Total N = 1387
Number of patients reported		125	130	117	41	413
Sex ^32^*	Men	84	44	90	26	244
	Women	36	40	52	7	137
Age ^(sd)4^		31.7 (12.2)	34.0 (10.7)	32.6 (11.6)	28.6 (12.3)	31.8 (12.2)

BPS: British Psychological Society; BACP: British Association for Counselling and Psychotherapy; UKCP: United Kingdom Council for Psychotherapy; RCP Royal College of Psychiatrists

Number in superscript – missing

* one transgender patient seen by a psychiatrist

### Services to modify a same-sex sexual orientation

The 222 professionals who had helped clients to change their sexual orientation then answered the following question: "Given the extent of knowledge about homosexuality and treatments available to change or redirect homosexual or lesbian feelings, are there any circumstances where people should have the opportunity to reduce or redirect their homosexual or lesbian feelings?" One hundred and fifty nine (72%) agreed and 28 (13%) disagreed with the statement, while 35 (15%) gave no answer. Those who answered affirmatively were then given an opportunity to explain more fully what such a service would mean. There was substantial overlap in the themes arising from the participants in the four professional groups. All groups considered that a client/patient's distress about their homosexuality was justification for intervention; they cited religious, cultural and moral values as likely causes for internal conflict:

"...where someone had a strong faith, then working to help the person accept their feelings but manage them appropriately may be the best approach if (the) person felt they would lose God and therefore their life was not worth living." (BACP).

"In many societies/cultures expression of sexuality out with cultural norms can cause huge distress. Given the balance between biological and developmental determinants of sexuality it is valid for an individual to value his cultural norms and to try and reduce the distress caused by transgressing these." (Psych).

"Yes, possibly those within marriages that wish to continue with that relationship rather than break up" (UKCP).

"People should be given the opportunity to choose to redirect their sexual feelings depending on their circumstances. For example the homosexual man I helped to become heterosexual came from a working class background where it was completely unacceptable to deviate from the norm. It was extremely important to him to be accepted by that community." (BPS).

"Client ultimately knows best and may have deep religious beliefs that influence them enormously." (BACP).

"The individuals I have worked with have all been very unhappy about their sexuality and wish they were heterosexual. This has been because of responses from friends, family and the local community – which outside London is still very homophobic." (BPS).

The wishes of the client/patient were mentioned by all groups of practitioners with self- determination being seen as an issue that might override a degree of professional unease:

"(the) client is 'the expert' and I deal with their realities rather than mine." (BACP).

"People should have the choice to explore change while at the same time the therapist can hold to their ethical stance." (UKCP).

"If after extensive, good therapy they were still adamant they wanted to change, I would think this was their decision though I would hope they would come to terms with themselves on the journey." (BACP).

"I feel people should have the opportunity to consider their sexuality and if they want to reduce or redirect any aspect of it they should be helped to do so." (BPS).

"We have a responsibility to assist our patients with self-determination." (Psych).

Professionals often perceived that their clients were confused about their sexual preference and it appeared that from a professional perspective bisexuality was not a stable category of sexual orientation:

"Some bisexual individuals may wish to choose an orientation that is comfortable for them and their lifestyle choices for example. This is a therapeutic issue to explore and support if that is their wish. It is different from behavioural attempts to reshape desire." (UKCP).

"I am sure there are cases of bisexuality or sexual ambivalence where counselling could be offered to motivated individuals." (Psych).

Confusion was seen as a particular issue in younger patients/clients:

"Because some clients/patients are unsure of whether they are really homosexual – particularly young adults under 25." (BPS).

"Children and young adults are more likely to be confused about their sexuality and to jump to conclusions (correct or otherwise) if unable to talk through their concerns." (Psych).

Occasionally professionals considered that a history of sexual abuse had possibly had an impact on sexual preference and for this reason clients wished to reduce or redirect their same sex attraction:

"Adult sexual preferences (either homo or heterosexual) may be confused or unsatisfying because of previous experiences such as childhood sexual abuse. Once early sexual trauma has been resolved the client may shift energy to more satisfying adult sexual relationships and may change sexual preferences." (UKCP).

Only the psychiatrists expressed scepticism about the likely outcome of any treatment to change sexual orientation. However, they were the only professionals who also suggested that a conviction about same sex attraction could be a feature of major mental illness that would respond to treatment:

"These feelings can arise in the context of psychotic illness where it can be difficult to be clear about the underlying sexuality." (Psych).

"I think sometimes sexual identity can become fragmented as part of a more general psychotic disorder. The treatment I would offer is to treat the psychotic illness and then assess the person's anxieties if still remaining re their sexuality." (Psych).

A very small number of those advocating intervention in this area had discernibly negative views about the same sex relationships:

"Although homosexual feelings are usual in people, their physical expression, and being a person's only way of having sexual relations is problematic. The physical act for male homosexuals is physically damaging and is the main reason in this country for AIDS/HIV. It is also perverse.........." (BPS).

Only 28 of the 222 professionals with experience of work in this area (see above) thought there were no circumstances in which individuals should have the opportunity to redirect or reduce their homosexual or lesbian feelings. Those who replied in this way emphasized the need both to explore people's sexuality rather than to change it:

"It is up to the person themselves to decide which direction to go in. I am just the sounding board for them to make their own decisions." (UKCP).

"It is better to help people look at the problems and come to a decision for themselves. If people are homosexual/lesbian that is what they are." (BPS).

"Helping them to clarify the situation is important." (Psych).

"I believe people with homosexual feelings sometimes need to use therapy to explore their feelings, to have a clear vision about their sexuality." (BACP).

"I would not assume I knew what direction someone should take." (BACP).

## Discussion

### Main findings

Our results indicate that asking therapists whether or not they would attempt to change a client's sexual orientation if requested, yielded very few (4%) who say they would do so. However, when asked about specific instances in the past, 17% of therapists reported having treated at least one patient in order to change their sexual orientation from homosexual to heterosexual and there was no sign of a decline of such treatments in recent years. Older male therapists and members of the BPS and UKCP were more likely to have done so. One third of clients treated were women, just over half were treated in private practice and most received some form of counselling. Beyond providing counselling, there appeared to be no consistent approach to treatment. Three quarters of these therapists considered that a service should be provided for gay and lesbian people who wanted to become heterosexual.

Key issues for these professionals included the role of external value systems (e.g. family, religious, social or ethical values) in determining a client/patient' distress and the right of individuals to determine the aim of their treatments. Although many of these descriptions were about patients and settings some time in the past when social norms in England and Wales were less inclusive of homosexuality, it is of concern that there seems to have been no reduction in such treatments over recent decades. When account is also taken of the ethical dilemmas posed by this work, it is difficult to see why a proportion of professionals continue to see this as a reasonable undertaking.

### Strengths and limitations

It was clear from the high response rates and their added comments that the questions provoked interest among the therapists. Despite the high response rate, however, it is possible that professionals who conduct or recommend therapy to change sexual orientation might choose not to respond and thus our figures may under-estimate the frequency of such practices. However, collecting such data by postal questionnaire limits the detail that can be explored and often results in missing data. It also relies on therapists' memories and the accuracy of information recalled in some cases over decades must be open to doubt. In addition, our data about the characteristics and motivations of clients comes only from the therapists' accounts. Furthermore, although three quarters of these patients were followed up by the therapists to assess outcome, we were unable to collect any further information on the nature of that outcome. Finally, we asked only the 222 therapists who had treated a client about the desirability of services to change people from homosexual to heterosexual. Although this might be seen as a limitation, we believe this was logical, because the question would have been hypothetical for the other therapists.

### The clients

Our data reveal that a substantial minority of women are treated to change same-sex desire, as opposed to the great preponderance of men described in the (mainly behaviourist) literature of the 1970s and 1980s [1]. They also reveal that although behavioural treatments now appear to be uncommon, therapists continue to provide other talking therapies to help clients to change.

### The efficacy and ethics of treatment

There is no evidence from the published literature to suggest that a person's sexual orientation can be changed from homosexual to heterosexual. Earlier forms of both psychoanalytic and behavioural research showed no evidence of efficacy [1] and many therapists later regretted their involvement in such treatments [2,4]. Furthermore, recent research into the effectiveness of so-called reparative therapy to change sexual orientation in the United States has demonstrated little evidence of efficacy [5,6] and considerable controversy [7] about the quality of the methods used. All have been post hoc evaluations of volunteers, often some years after receipt of treatment. All fall well short of the quality guidelines for outcome research [8]. Furthermore, the American Psychiatric Association is directly opposed to 'any psychiatric treatment, such as "reparative" or conversion therapy, which is based upon the assumption that homosexuality per se is a mental disorder or based upon the a priori assumption that a patient should change his/her sexual homosexual orientation' [9].

Thus, it is hard either to understand or recommend the actions of the one in six psychotherapists, counsellors and psychiatrists who undertook these treatments. The qualitative data suggest that they made therapeutic decisions based on privileging client/patient choice where there was a wish to avoid the impact of negative social attitudes to same sex relationships. They appeared to take little account of the potential harm of applying treatments with no evidence for efficacy. Furthermore, the commonest reason for the referral was confusion about sexual orientation rather than an expressed desire to change it. It is well known that confusion is both a feature of a developmental trajectory, often part of the "coming out" story, and a common reason for seeking help [10,11]. It appears unlikely that therapists were responding straightforwardly to the demands of patients as direct requests for change were very rarely reported. However, there is some evidence that the satisfaction of gays and lesbians with mental health services, and with psychotherapy in particular, may be improving. There is a well-known body of work documenting discriminatory approaches to gay and lesbian clients in health services in general [12] and mental health in particular [13-15]. However, more recent work has suggested that gays and lesbians may be more satisfied than heterosexuals with the service they receive [16,17] and that repeated contact can result in confirmation of a positive gay and lesbian identity [11,18]. The fluidity of some therapeutic approaches and an implicit theoretical framework, rather than explicit diagnostic or behavioural approaches, may allow for this. It is also possible that contemporary practitioners, when faced with someone in their clinic, do indeed follow the client rather than the textbook.

## Conclusion

Treatments to change sexual orientation do not appear to have become completely a thing of the past. Guidelines on appropriate approaches to clients who are confused or upset about same-sex desires could be useful as a reliance on clinicians' inherent attitudes may still leave the door open to discrimination [19], which in gays and lesbians is itself linked with psychological distress [20].

## Abbreviations

BPS: British Psychological Society; BACP: British Association for Counselling and Psychotherapy; UKCP: United Kingdom Council for Psychotherapy; RCP: Royal College of Psychiatrists.

## Competing interests

The authors declare that they have no competing interests.

## Authors' contributions

MK and AB conceived the idea for the study and obtained grant funding. GS carried out the survey. MK analysed the quantitative and AB the qualitative data.

## Acknowledgements

This research was funded by a Wellcome Trust History of Medicine Grant.

## Pre-publication history

The pre-publication history for this paper can be accessed here:


